# The Effects of Fetal Gender on Maternal and Fetal Insulin Resistance

**DOI:** 10.1371/journal.pone.0137215

**Published:** 2015-09-14

**Authors:** Jennifer M. Walsh, Ricardo Segurado, Rhona M. Mahony, Michael E. Foley, Fionnuala M. McAuliffe

**Affiliations:** 1 UCD Obstetrics and Gynaecology, School of Medicine and Medical Science, University College Dublin, National Maternity Hospital, Dublin, Ireland; 2 CSTAR, School of Public Health, Physiotherapy and Population Science, University College Dublin, Dublin, Ireland; 3 National Maternity Hospital, Dublin, Ireland; The University of Manchester, UNITED KINGDOM

## Abstract

**Objective:**

Gender plays a role in the development of a number of cardiovascular and metabolic diseases and it has been suggested that females may be more insulin resistant in utero. We sought to assess the relationship between infant gender and insulin resistance in a large pregnancy cohort.

**Study Design:**

This is a secondary analysis of a cohort from the ROLO randomized control trial of low GI diet in pregnancy. Serum insulin, glucose and leptin were measured in early pregnancy and at 28 weeks. At delivery cord blood C-peptide and leptin were measured. A comparison of maternal factors, fetal biometry, insulin resistance and leptin was made between male and female offspring. A multivariate regression model was built to account for the possible effects of maternal BMI, birthweight and original study group assignment on findings.

**Results:**

A total of 582 women were included in this secondary analysis, of whom 304 (52.2%) gave birth to male and 278 (47.8%) gave birth to female infants. Compared to male infants at birth, female infants were significantly lighter, (3945 ± 436 vs. 4081± 549g, p<0.001), shorter in length (52.36 ± 2.3 vs. 53.05 ± 2.4cm, p<0.001) and with smaller head circumferences (35.36 ± 1.5 vs. 36.10 ± 1.1cm, p<0.001) than males. On multiple regression analysis, women pregnant with female fetuses were less insulin resistant in early pregnancy, i.e. had lower HOMA indices (B = -0.19, p = 0.01). Additionally female fetuses had higher concentrations of both cord blood leptin and C-peptide at birth when compared to male offspring (B = 0.38, p<0.001 and B = 0.31, p = 0.03 respectively).

**Conclusion:**

These findings suggest gender is a risk factor for insulin resistance in–utero. Additionally, carrying a female fetus decreases the risk of insulin resistance in the mother, from as early as the first trimester.

## Introduction

Significant sex differences exist in the propensity to develop metabolic dysfunction and, in particular, type 2 diabetes [1–2]. In adult populations, the prevalence of diabetes is nearly twice as high in men as in women [2], and coronary heart disease is consistently associated with greater mortality rates and risks in male compared to female populations [3]. In younger populations, however, a contrasting gender association is becoming apparent. Measurements of insulin resistance in both children and adolescents from a variety of ethnic backgrounds have been demonstrated to be higher in girls when compared to boys [4–7]. This gender disparity may be assumed to reflect genetic variations in fat and fat free mass, which may not translate to clinically relevant differences; however it is increasingly evident that this is unlikely to be the case. The rising prevalence of obesity worldwide has been accompanied by an increase in the incidence of type 2 diabetes in younger populations [8, 9]. Interestingly, it is being increasingly and consistently demonstrated that among this group, the incidence of the disease in females is higher than that in males [7–9].Most recently evidence is emerging that this gender disparity may be programmed in utero. Female newborns have been shown to have higher insulin concentrations at birth than male newborns, despite being smaller, suggesting intrinsic insulin resistance in girls [10, 11]. It has also been suggested that during pregnancy, a mother’s degree of insulin resistance may be influenced by the gender of the fetus she is carrying, though these data are inconsistent in the existing literature. Though one recent paper suggested that pregnancy with a female fetus is associated with greater maternal insulin resistance at 24 to 28 weeks gestation [12], a number of larger and population based studies have suggested the opposite; that women carrying a male fetus have a higher likelihood of developing gestational diabetes [13–15].We recently conducted a randomised control trial of low glycaemic index diet in pregnancy for the prevention of recurrence of macrosomia [16,17]. This trial, the ROLO study, included a large cohort of euglycaemic women who had previously given birth to infants weighing greater than 4000g. As part of this study, assessments of fetal adiposity and neonatal anthropometry were recorded, and longitudinal assessments of maternal and fetal insulin resistance, and leptin concentrations, were performed. These assessments have enabled us to further probe the role of gender in the programming of obesity and metabolic dysfunction, by examining the relationships between infant gender and fetal adiposity, insulin resistance and leptin in this large, well-characterised pregnancy cohort.

## Materials and Methods

### Study sample

This is a secondary analysis of a cohort of women and infants from the ROLO randomized control trial. ROLO was a trial of low glycaemic index diet in pregnancy versus no dietary intervention for the prevention of macrosomia. Women were recruited in early pregnancy at first antenatal consultation at a mean gestational age of 12.9 ± 3.0 weeks. As per the study protocol, all women were secundigravid having previously delivered an infant weighing greater than 4000g. Women were excluded if they had pre-existing or previous gestational diabetes. We also excluded those <18 years of age and those unable to give full informed consent. For the purposes of this analysis, women with laboratory assessments of insulin resistance (n = 582/781, 75%) were included. There were no differences in the baseline characteristics between those who did and those who did not have laboratory analyses performed. Detailed methodology of the ROLO study has been previously published [16, 17]. The controlled trial was registered as ISRCTN54392969.

### Ethics statement

The ROLO study was approved by the Ethics Committee of the National Maternity Hospital (June 2006); written informed consent was obtained.

### Assessments

At first antenatal consultation (baseline), women had their height and weight recorded and body mass index (BMI) calculated. Women were then randomly allocated to low glycaemic index (GI) dietary advice or routine care. At this visit and again at 28 weeks gestation fasting serum glucose, insulin, and leptin concentrations were measured. Fetal ultrasound at 34 weeks gestation assessed fetal biometry including estimated fetal weight (EFW), abdominal circumference (AC) and fetal adiposity (anterior abdominal wall width, AAW). AAW was measured in the traditional abdominal circumference view and included fetal skin and subcutaneous tissue. Three measurements were taken and the mean recorded. This measurement has been used in populations both with and without diabetes as a marker of fetal adiposity [18,19]. One of two sonographers performed the ultrasound measurements using a Voluson 730 Expert (GE Medical Systems, Germany). At delivery, we recorded infant birth weight, gestational age (days), infant length, head circumference and placental weight. Cord blood concentrations of fetal leptin and C-peptide were also measured.

### Laboratory Methods

Multianalyte profiling was performed on the Luminex Magpix system (Luminex Corporation). Plasma concentrations of leptin, insulin and C-peptide were determined by the Human Endocrine Panel. Maternal insulin resistance was calculated using the homeostasis model assessment (HOMA) index [20, 21]: HOMA score = (fasting insulin μUml^-1^ x fasting glucose mmoll^-1^)/22.5. Fetal insulin resistance was assessed by cord blood C-peptide estimation. Cord blood haemolysis occurred in approximately 17% of samples prohibiting analysis of leptin and C-peptide in these cases.

### Statistical Analysis

A comparison of maternal factors, fetal biometry, insulin resistance and leptin was made between male and female offspring. Data were assessed for normality using Shapiro-Wilk and PP plot and visualisation of histograms. Skewed data were log transformed prior to analysis. A multivariate regression model was built to account for the possible effects of maternal BMI, birthweight and original study group assignment on findings. Statistical analysis was performed using SPSS version 21.0 (IBM, Armonk, NY, USA). A two-tailed *P* value of less than 0.05 was considered significant.

## Results

A total of 582 women were included in this secondary analysis, of whom 304 (52.2%) gave birth to male and 278 (47.8%) gave birth to female infants. The baseline characteristics of the cohort are contained in Table 1. The mean BMI of the group was 26.8 ± 4.9kg/m^2^ in early pregnancy and the mean gestational age at delivery was 281 days for each gender. There were no differences in maternal characteristics at baseline between those who subsequently delivered male or female infants. Overall the mean birthweight at delivery was 4016 ± 502g. Compared to male infants, females in utero had lower EFW at 34 weeks, (2569 ± 347 vs. 2663 ± 340g, p = 0.01) and smaller AC’s (313 ± 18.8 vs. 316 ± 16.9mm, p = 0.03). Despite these differences, no difference in abdominal wall fat (AAW) was observed (5.04 ± 1.1 vs. 5.06 ± 1.3mm, p = 0.8). At birth, female infants were significantly lighter, (3945 ± 436 vs. 4081± 549g, p<0.001), shorter in length (52.36 ± 2.3 vs. 53.05 ± 2.4cm, p<0.001) and with smaller head circumferences (35.36 ± 1.5 vs. 36.10 ± 1.1cm, p = 0.001) than males. Placental weights were also lighter in females (752.9 ± 163 vs. 787.9 ± 176g, p = 0.01). Table 2. All women included in this analysis had early pregnancy HOMA indices measured (n = 582.) Early pregnancy leptin was available in 98.1% (n = 571, 297 male and 274 female). Leptin at 28 weeks was available in 95.3% of the cohort (n = 555, 288 male and 267 female), and the HOMA index in 95.5% at the same time (n = 556, 288 male and 268 female). In cord blood at delivery C-peptide results were obtained in 82.5% of the cohort (n = 480, 254 male and 226 female) and leptin in 80.9% (n = 471, 248 male and 223 female). On simple unadjusted analysis, women pregnant with female offspring had lower insulin resistance (HOMA) in early pregnancy. At delivery, female infants had higher concentrations of cord blood leptin when compared to males, with a trend toward higher cord C-peptide concentrations also. Table 3. Following adjustment for maternal early pregnancy BMI, birthweight, smoking and original study group assignment on multiple regression analysis three significant differences between male and female offspring were apparent. Women pregnant with female fetuses were less insulin resistant in early pregnancy, i.e. had lower HOMA indices (B = -0.19, p = 0.01). Additionally female fetuses had higher concentrations of both cord blood leptin and cord blood C-peptide when compared to male offspring (B = 0.38, p<0.001 and B = 0.31, p = 0.03 respectively). Table 3.

**Table 1:** Baseline characteristics.

Baseline characteristic	Overall	Female fetus	Male fetus	P-value
BMI (kg/m^2^)	26.78 (4.9)	26.35 (4.6)	27.17 (5.1)	0.05
Early pregnancy glucose (mmol\l)	4.41 (0.3)	4.37 (0.3)	4.44 (0.3)	0.06
Smoking (n)	31	14	17	0.7
Gestation at delivery (days)	281.53 (9.7)	281.62 (8.6)	281.43 (10.6)	0.7

Data presented as mean and standard deviations or N. BMI is body mass index as calculated at first antenatal consultation. A p-value of less than 0.05 considered statistically significant.

**Table 2:** A comparison of fetal and neonatal anthropometry between female and male offspring.

	Female	Male	P-value
EFW (grams)	2569 (347)	2663 (340)	0.01*
AC (mm)	313 (18.8)	316 (16.90)	0.03*
AAW (mm)	5.04 (1.1)	5.06 (1.3)	0.8
Birthweight (grams)	3945 (436)	4081 (549)	<0.001*
Length at birth (cm)	52.36 (2.3)	53.05 (2.4)	<0.001*
Head circumference at birth (cm)	35.36 (1.5)	36.10 (1.1)	0.001*
Placental weight (grams)	752.9 (163)	787.9 (176)	0.01*

Data presented as mean and standard deviations. EFW is estimated fetal weight, AC is abdominal circumference and AAW is anterior abdominal wall width, all at 34 weeks.

* A p-value of less than 0.05 considered statistically significant.

**Table 3:** A comparison of maternal and fetal insulin resistance and leptin between female and male offspring.

	Female		Male		Crude p-value	Beta	Adjusted p-value	95% CI for Beta
	Mean ± SD	Median	Mean ± SD	Median				
Early pregnancy HOMA	1.66±1.14	1.14	2.34±3.0	1.69	0.002	-0.19	0.011*	-0.34,-0.05
Early pregnancy leptin (pg/ml)	15250.81±625.62	12240.81	17280.84±19702.78	13868.31	0.710	0.07	0.274	-0.06,+0.19
HOMA at 28 weeks	2.32±2.99	1.77	3.04±4.97	2.05	0.067	-0.09	0.217	-0.24,+0.06
Leptin at 28 weeks (pg/ml)	19713.37±15056.77	16401.46	20327.68±15046.76	16682.04	0.816	0.02	0.741	-0.11,+0.16
Cord blood C-peptide (pg/ml)	562.82±698.01	259.76	454.65±639.71	164.68	0.061	0.31	0.027*	+0.04,+0.59
Cord blood leptin (pg/ml)	29768.54±23140.75	24017.33	24094.15±20468.04	18669.77	0.001	0.38	<0.001*	+0.23,+0.53

Data presented as mean and standard deviations and median. Crude p-value derived from students T-test of log transformed data. Beta represents strength and direction of adjusted relationship. Adjusted p-value adjusted for maternal BMI, infant birthweight, smoking status and original study group assignment with multiple regression analysis. Regression coefficients (beta) based on log-transformed data.

* A p-value of less than 0.05 considered statistically significant.

## Discussion

Our findings have confirmed that the female fetus is more insulin resistant in-utero with higher leptin and C-peptide concentrations in cord blood despite weighing less at birth. These findings are in keeping with the recent growing body of evidence suggesting that in both childhood and adolescence, girls are intrinsically more insulin resistant than boys. Among overweight and obese African American 5–10 year olds, girls were more likely to be insulin resistant than boys, already demonstrating early signs of metabolic decompensation in response to a glucose load [4]. Similar findings were confirmed in the Early Bird cohort study of over 300 healthy children. The authors found in their cohort at 5 years of age, girls were intrinsically more insulin resistant than boys. Insulin resistance was 35% higher in girls than in boys. Girls carried 26% more subcutaneous fat despite similar body weights. However, after correcting for anthropometric variables and physical activity, girls remained 33% more insulin resistant than boys [7]. Studies such as ours, which involve assessments of cord blood and neonatal anthropometry at birth, provide evidence that this increased tendency toward insulin resistance in females may be genetically rather than environmentally dictated. To date there are a small number of studies which confirm our findings [10, 11]. Our cohort of over 500, well characterised healthy pregnant women is larger than previous studies, and our findings are adjusted to account for possible confounders, in particular the possible effects of the study intervention, maternal BMI and infant birthweight. This adjustment for birthweight is of particular importance in any study involving infant gender, as female infants have a tendency to weigh less at birth than their male counterparts. In this instance simple non-adjusted analyses, resulted in identical conclusions to the adjusted findings. The addition of leptin adds biological plausibility to our findings in relation to insulin resistance. Leptin is an adipocytokine involved in satiety and energy regulation through appetite control [22]. Elevated plasma leptin levels are observed in obesity and insulin resistance [23]. Fetal leptin is derived primarily from fetal adipose tissue, with 98% of placental leptin entering the maternal circulation [24], making cord blood leptin levels particularly representative of levels in intrauterine life. Despite the higher concentrations of cord leptin in females, we did not identify a corresponding gender difference in fetal adiposity, as might have been expected. At 34 weeks, males and females had similar anterior abdominal wall widths, albeit that males were larger, and therefore may have been expected to have more adiposity. Fetal fat deposition is maximal in the later stages of pregnancy and as such, there may have been differences in fat deposition between males and females at birth that were not apparent at our 34-week assessment. It is undoubtedly a limitation that neonatal fat mass was not assessed as part of this study. Nonetheless, ultrasound examination is a validated method for measurement of fetal subcutaneous tissue, and has been shown to be predictive of mechanical neonatal skinfold thickness measurements [25]. These findings have potential implications for clinical practice. The rising prevalence of type 2 diabetes, traditionally considered a disease of later adulthood, in childhood and adolescence, is an increasing public health concern [26]. It is becoming clear that when type 2 diabetes does occur in childhood, it is significantly more common in girls than boys. The knowledge that this increased tendency may be programmed from early fetal life might suggest that any interventions to reduce its incidence, such as the encouragement of regular physical activity and a healthy diet should be instituted early in life, particularly in girls. A number of studies have identified that girls are less likely to engage in regular exercise in childhood than males [27,28]. These findings would suggest that efforts should be made to reverse this tendency. Our findings in relation to maternal insulin resistance in early pregnancy are particularly interesting as this is a source of recent conflicting reports in the literature. We are aware of one recent study that did identify, for the first time, that the female fetus is associated with greater maternal insulin resistance at 24 to 28 weeks [12]. In contrast, we have identified that carrying a female fetus is protective of insulin resistance as early as the first and second trimester. Our findings are in keeping with a recent, large retrospective study of over 600,000 births, which confirmed that carrying a boy increased the risk of maternal gestational diabetes in both first and second pregnancies [13]. The link between a male gender fetus and maternal insulin resistance has been suggested previously [14, 15], but to the best of our knowledge, ours is the first to establish that the link between maternal insulin resistance and infant gender may be established as early as the first trimester of pregnancy. There are a number of hypotheses for this potential gender effect. A number of placentally derived hormones may affect maternal insulin resistance, in particular, placental lactogen and oestriol, which may be increased with a larger placental mass[29,30]. This gender difference in maternal insulin resistance was not identified at 28 weeks. Though women carrying male offspring did have higher HOMA indices at 28 weeks, this failed to reach statistical significance. As a secondary analysis of the original ROLO randomised control trial, this secondary analysis may have been underpowered to detect a difference in insulin resistance at this time-point. There is a need for further studies to interrogate this relationship between fetal gender and both fetal and maternal insulin resistance further. The ROLO study protocol [16] dictated that all women in our cohort had previously given birth to a macrosomic infant, and half were subject to low GI dietary advice. Though we adjusted for both birthweight and study group assignment in our analyses, the results need to be validated in a cohort of women giving birth to infants with a normal range of birthweight. Our cohort was euglycaemic, with no previous history of gestational diabetes, which likely limited our ability to establish a link between later pregnancy insulin resistance and infant gender. Though our results have suggested a clear link between gender and intrinsic insulin resistance, further work is needed to probe the underlying cause. There may be variations in placental hormones or genetic predispositions in particular females that increase sensitivities toward insulin resistance, rather than a predisposition in all. Follow-up studies of this and similar cohorts may in time reveal if statistical differences in leptin and insulin resistance translate into clinically meaningful outcomes.

In conclusion, the present study adds to the growing body of evidence linking female gender and the prenatal programming of intrinsic insulin resistance. It also adds the novel suggestion that the female fetus confers an decreased risk of insulin resistance to the mother from an early gestation. Further work is now needed to validate these findings in a more generalised population, and to determine the potential clinical implications for childhood and adult health.

## References

[1] Perreault L, Ma Y, Dagogo-Jack S, Horton E, Marrero D, Crandall J, et al Diabetes Prevention Program. Sex differences in diabetes risk and the effect of intensive lifestyle modification in the Diabetes Prevention Program. Diabetes Care. 2008 7;31(7):1416–21. 10.2337/dc07-2390 18356403 PMC2453677

[2] Barrett-Connor E. The Rancho Bernardo Study: 40 years studying why women have less heart disease than men and how diabetes modifies women's usual cardiac protection. Glob Heart. 2013 6 1;8(2) 10.1016/j.gheart.2012.12.002 PMC3810980 24187655

[3] Barrett-Connor E. Gender differences and disparities in all-cause and coronary heart disease mortality: epidemiological aspects. Best Pract Res Clin Endocrinol Metab. 2013 8;27(4):481–500. 10.1016/j.beem.2013.05.013 24054926 PMC3781943

[4] Young-Hyman D, Schlundt DG, Herman L, De Luca F, Counts D. Evaluation of the insulin resistance syndrome in 5- to 10-year-old overweight/obese African-American children. Diabetes Care. 2001;24:1359–1364. 11473070 10.2337/diacare.24.8.1359

[5] Bavdekar A, Yajnik CS, Fall CH, Bapat S, Pandit AN, Deshpande V, et al Insulin resistance syndrome in 8-year-old Indian children: small at birth, big at 8 years, or both? Diabetes 1999 12;48(12):2422–9. 10580432 10.2337/diabetes.48.12.2422

[6] Fagot-Campagna A, Pettitt DJ, Engelgau MM, Burrows NR, Geiss LS, Valdez R, et al Type 2 diabetes among North American children and adolescents: an epidemiologic review and a public health perspective. J Pediatr. 2000 5;136(5):664–72. 10802501 10.1067/mpd.2000.105141

[7] Murphy MJ, Metcalf BS, Voss LD, Jeffery AN, Kirkby J, Mallam KM, et al Girls at five are intrinsically more insulin resistant than boys: The Programming Hypotheses Revisited—The EarlyBird Study (EarlyBird 6). Pediatrics. 2004 1;113(1 Pt 1):82–6. 14702453 10.1542/peds.113.1.82

[8] Pettitt DJ, Talton J, Dabelea D, Divers J, Imperatore G, Lawrence JM, et al SEARCH for Diabetes in Youth Study Group. Prevalence of diabetes in U.S. youth in 2009: the SEARCH for diabetes in youth study. Diabetes Care. 2014 2;37(2):402–8. 10.2337/dc13-1838 24041677 PMC3898760

[9] Ehtisham S, Hattersley AT, Dunger DB, Barrett TG; British Society for Paediatric Endocrinology and Diabetes Clinical Trials Group. First UK survey of paediatric type 2 diabetes and MODY. Arch Dis Child. 2004 6;89(6):526–9. 15155395 10.1136/adc.2003.027821 PMC1719947

[10] Shields BM, Knight B, Hopper H, Hill A, Powell RJ, Hattersley AT, et al Measurement of cord insulin and insulin-related peptides suggests that girls are more insulin resistant than boys at birth. Diabetes Care 2007; 30: 2661–2666. 17475939 10.2337/dc06-1501

[11] Ibáñez L, Sebastiani G, Lopez-Bermejo A, Díaz M, Gómez-Roig MD, de Zegher F. Gender specificity of body adiposity and circulating adiponectin, visfatin, insulin, and insulin growth factor-I at term birth: relation to prenatal growth. J Clin Endocrinol Metab. 2008 7;93(7):2774–8. 10.1210/jc.2008-0526 18460569

[12] Xiao L, Zhao JP, Nuyt AM, Fraser WD, Luo ZC. Female fetus is associated with greater maternal insulin resistance in pregnancy. Diabet Med. 2014 12;31(12):1696–701. 10.1111/dme.12562 25112731

[13] Retnakaran R, Shah BR. Fetal Sex and the Natural History of Maternal Risk of Diabetes During and After Pregnancy. J Clin Endocrinol Metab. 2015 5 20:jc20151763. [Epub ahead of print] 10.1210/jc.2015-1763 25993641

[14] Retnakaran R, Kramer CK, Ye C, Kew S, Hanley AJ, Connelly PW, et al Fetal sex and maternal risk of gestational diabetes mellitus: the impact of having a boy. Diabetes Care. 2015 5;38(5):844–51. 10.2337/dc14-2551 25693837

[15] Sheiner E, Levy A, Katz M, Hershkovitz R, Leron E, Mazor M. Gender does matter in perinatal medicine. Fetal Diagn Ther. 2004;19(4):366–369. 15192298 10.1159/000077967

[16] Walsh J, Mahony R, Foley M, McAuliffe F. A randomised control trial of low glycaemic index carbohydrate diet versus no dietary intervention in the prevention of recurrence of macrosomia. BMC Pregnancy Childbirth. 2010 4 23;10:16 10.1186/1471-2393-10-16 20416041 PMC2876071

[17] Walsh JM, McGowan CA, Mahony R, Foley ME, McAuliffe FM. Low glycaemic index diet in pregnancy to prevent macrosomia (ROLO study): randomised control trial. BMJ. 2012 8 30;345:e5605 10.1136/bmj.e5605 22936795 PMC3431285

[18] Higgins MF, Russell NM, Mulcahy CH, Coffey M, Foley ME, McAuliffe FM. Fetal anterior abdominal wall thickness in diabetic pregnancy. Eur J Obstet Gynecol Reprod Biol 2008;140:43–7. 10.1016/j.ejogrb.2008.02.021 18406510

[19] Petrikovsky BM, Oleschuk C, Lesser M, Gelertner N, Gross B. Prediction of fetal macrosomia using sonographically measured abdominal subcutaneous tissue thickness. J Clin Ultrasound 1997;25:378–82. 9282803 10.1002/(sici)1097-0096(199709)25:7<378::aid-jcu5>3.0.co;2-7

[20] Matthews DR, Hosker JP, Rudenski AS, Naylor BA, Treacher DF, Turner RC. Homeostasis model assessment: insulin resistance and beta-cell function from fasting plasma glucose and insulin concentrations in man. Diabetologia. 1985 7;28(7):412–9. 3899825 10.1007/BF00280883

[21] Bonora E, Targher G, Alberiche M, Bonadonna RC, Saggiani F, Zenere MB, et al Homeostasis model assessment closely mirrors the glucose clamp technique in the assessment of insulin sensitivity. Diabetes Care. 2000;23:57–63. 10857969 10.2337/diacare.23.1.57

[22] Stofkova A. Leptin and adiponectin: from energy and metabolic dysbalance to inflammation and autoimmunity. Endocr Regul. 2009;43:157–68. 19908934

[23] Levi J, Gray SL, Speck M, Huynh FK, Babich SL, Gibson WT, et al Acute disruption of leptin signaling in vivo leads to increased insulin levels and insulin resistance. Endocrinology. 2011 9;152(9):3385–95. 10.1210/en.2011-0185 21750049

[24] Linnemann K, Malek A, Sager R, Blum WF, Schneider H, Fusch C. Leptin production and release in the dually in vitro perfused human placenta. J Clin Endocrinol Metab. 2000 11;85(11):4298–301. 11095471 10.1210/jcem.85.11.6933

[25] Buhling KJ, Doll I, Siebert G, Catalano PM. Relationship between sonographically estimated fetal subcutaneous adipose tissue measurements and neonatal skinfold measurements. Ultrasound Obstet Gynecol. 2012 5;39(5):558–62. 10.1002/uog.10092 21898636

[26] Dabelea D, Mayer-Davis EJ, Saydah S, Imperatore G, Linder B, Divers J, et al SEARCH for Diabetes in Youth Study. Prevalence of type 1 and type 2 diabetes among children and adolescents from 2001 to 2009. JAMA. 2014 5 7;311(17):1778–86. 10.1001/jama.2014.3201 24794371 PMC4368900

[27] Telama R, Yang X, Leskinen E, Kankaanpää A, Hirvensalo M, Tammelin T, et al Tracking of physical activity from early childhood through youth into adulthood. Med Sci Sports Exerc. 2014;46(5):955–62. 10.1249/MSS.0000000000000181 24121247

[28] Fakhouri TH, Hughes JP, Burt VL, Song M, Fulton JE, Ogden CL. Physical activity in U.S. youth aged 12–15 years, 2012. NCHS Data Brief. 2014 1;(141):1–8. 24401547

[29] Houghton DJ, Shackleton P, Obiekwe BC, Chard T. Relationship of maternal and fetal levels of human placental lactogen to the weight and sex of the fetus. Placenta. 1984 Sep-Oct;5(5):455–8. 6522356 10.1016/s0143-4004(84)80026-0

[30] Toriola AT, Vääräsmäki M, Lehtinen M, Zeleniuch-Jacquotte A, Lundin E, Rodgers KG, et al Determinants of maternal sex steroids during the first half of pregnancy. Obstet Gynecol. 2011 11;118(5):1029–36. 10.1097/AOG.0b013e3182342b7f 22015870 PMC3207141

